# CCL2 is required for initiation but not persistence of HIV infection mediated neurocognitive disease in mice

**DOI:** 10.1038/s41598-023-33491-7

**Published:** 2023-04-21

**Authors:** Boe-Hyun Kim, Eran Hadas, Jennifer Kelschenbach, Wei Chao, Chao-Jiang Gu, Mary Jane Potash, David J. Volsky

**Affiliations:** 1 Division of Infectious Diseases, Department of Medicine, Icahn School of Medicine at Mount Sinai, 1468 Madison Avenue, New York, NY 10029 USA; 2 Present Address: College of Life and Health Sciences, Institute of Biology and Medicine, Wuhan University of Science and Technology, Wuhan, Hubei China

**Keywords:** Biological techniques, Microbiology, Molecular biology, Neuroscience

## Abstract

HIV enters the brain within days of infection causing neurocognitive impairment (NCI) in up to half of infected people despite suppressive antiretroviral therapy. The virus is believed to enter the brain in infected monocytes through chemotaxis to the major monocyte chemokine, CCL2, but the roles of CCL2 in established NCI are not fully defined. We addressed this question during infection of conventional and CCL2 knockout mice with EcoHIV in which NCI can be verified in behavioral tests. EcoHIV enters mouse brain within 5 days of infection, but NCI develops gradually with established cognitive disease starting 25 days after infection. CCL2 knockout mice infected by intraperitoneal injection of virus failed to develop brain infection and NCI. However, when EcoHIV was directly injected into the brain, CCL2 knockout mice developed NCI. Knockout of CCL2 or its principal receptor, CCR2, slightly reduced macrophage infection in culture. Treatment of mice prior to and during EcoHIV infection with the CCL2 transcriptional inhibitor, bindarit, prevented brain infection and NCI and reduced macrophage infection. In contrast, bindarit treatment of mice 4 weeks after infection affected neither brain virus burden nor NCI. Based on these findings we propose that HIV enters the brain mainly through infected monocytes but that resident brain cells are sufficient to maintain NCI. These findings suggest that NCI therapy must act within the brain.

## Introduction

Combination antiretroviral therapy (CART) in people living with HIV (PLWH) suppresses HIV replication, prevents immunodeficiency, and extends lifetimes of people on CART. Nevertheless, other chronic ailments caused by HIV continue^1,2^. Among these, the predominantly mild HIV-associated neurocognitive disorders (HAND) occur in roughly half of CART compliant patients impairing day-to-day activities and reducing the quality of life^3^. With aging, PLWH are facing the fact that HAND tends to worsen with age^4^. To control or eliminate HAND, we must understand its viral and cellular bases.

In this effort, we have employed infection of conventional mice by EcoHIV, a chimeric HIV which encodes the ecotropic murine leukemia virus (MLV) envelope protein gp80 in place of gp120 thereby switching viral tropism from human to rodent^5^. EcoHIV largely reproduces HIV cell and tissue tropism, and systemic EcoHIV infection of conventional mice resembles chronic suppressed HIV infection in PLWH. However, EcoHIV infection does not induce immunodeficiency, possibly because the virus does not encode gp120^5^. We and others have shown that EcoHIV, like HIV, replicates primarily in CD4^+^ T lymphocytes, macrophages, and the brain; the virus establishes a lifelong chronic infection; and it induces several diseases seen in CART compliant PLWH, including neurocognitive impairment (NCI) and depression-like defects in all infected animals^5–15^. EcoHIV persistence in conventional mice correlates, as in PLWH, with low levels of latent/inducible provirus in CD4^+^ T lymphocytes and expressed virus in macrophages^15^. CD4^+^ T cells are the only cell type infected in spleen^5^ while thymocytes, the main cellular targets for MLV in mice, are not susceptible to EcoHIV infection in vivo^15^. Intracranial injection of EcoHIV into mice revealed that the virus mainly infects macrophage/microglial cells and not neurons in the brain^7,9^, a result reproduced using RNAscope in EcoHIV infected rats^11^. The control of EcoHIV replication in mice was attributed to both innate^7,16^ and HIV specific CD8^+^ T cell immune responses^17^. Other research suggests that EcoHIV, like HIV, can infect neuronal progenitor cells^18^ and pericytes^19^ in the central nervous system.

In EcoHIV-infected mice, as in other animal models and CART compliant PLWH themselves, macrophages and brain tissues express elevated levels of inflammatory cytokines, a spectrum of host antiviral factors, and chemokines that induce cellular migration^7,9,10,20,21^. Prominent in the latter category is CCL2, the primary chemokine for monocytes and an autocrine factor in HIV brain infection^22^. It is elevated in cerebrospinal fluid (CSF) of some individuals within weeks of HIV infection^20^. In macaque models of SIV encephalitis, elevated CCL2 in CSF compared to plasma predicts the development of disease^23^. Its synthesis is induced by viral gp120, Tat, Nef, and Vpr proteins and by interaction of astrocytes with HIV-infected macrophages^24–27^. Conversely, CCL2 promotes HIV infection in T cells and macrophages^28,29^. Moreover, in culture models, CCL2 has been shown to prompt transit of monocytes, and preferentially HIV-infected monocytes, across the blood–brain–barrier^30–32^. PLWH with a naturally occurring promoter variant in which CCL2 expression is increased show increased risks of HIV-associated dementia^33^ and their levels of CCL2 in the CSF correlate with the extent of impairment in cognitive tests^34^. Here we investigate the partnership of CCL2 and monocytes/macrophages in NCI during experimental EcoHIV infection of wildtype and CCL2 knockout (CCL2KO) mice or wildtype mice treated with CCL2 synthesis inhibitor, bindarit^35^. Our studies suggest that CCL2 is required for HIV entry into the brain and the development of NCI during systemic HIV infection but not after brain infection and disease have been established.

## Results

### Kinetics and dose response of EcoHIV brain disease development

**Figure 1:**
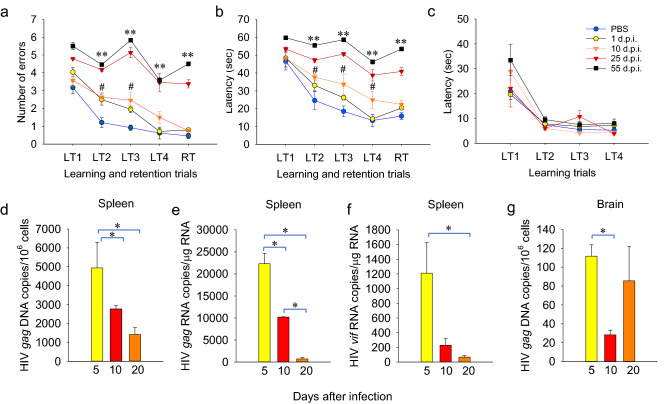
Kinetics of HIV-NCI development in EcoHIV infected C57BL/6 mice. Male 6–8-week-old C57BL/6 mice were infected with EcoHIV by IP injection and RAWM was performed 1, 10, 25, or 55 days later (n = 8 per condition/testing time post-infection, repeated once). Learning and short-term memory were measured by (**a**) mean number of errors ± standard errors of the mean (SEM) and (**b**) mean time ± SEM to find the hidden platform. (**c**) mean time ± SEM to find visible platform. LT1-LT4 denote acquisition trials and RT denotes the retention trial performed after a 30 min delay. RM ANOVA: (**a**) F(4,115) = 5.805, p < 0.001/LT2; F(4,115) = 10.163, p < 0.001/LT3; F(4,115) = 13.715, p < 0.001/LT4; (**b**) F(4,115) = 5.310, p < 0.001/LT2; F(4,115) = 16.793, p < 0.001/LT3; F(4,115) = 20.639, p < 0.001/LT4. For RT values, see text. Pairwise comparison by Student’s t-test. PBS *vs.* EcoHIV 25 and 55 days after infection, **p < 0.01; PBS *vs*. EcoHIV 10 days after infection, ^#^p < 0.05. (**d**–**g**) The experiment was repeated measuring EcoHIV burdens by QPCR in mice 5, 10, and 20 days after infection (n = 12 per group). (**d**) spleen HIV *gag* DNA, (**e**) spleen HIV *gag* RNA, (**f**) spleen HIV *vif* RNA, (**g**) brain HIV *gag* DNA. Pairwise comparison by t-test, *p < 0.05.

We first tested the time-course of EcoHIV brain infection and NCI to interrogate HIV brain entry in mice, a possible CCL2 function^22^. We infected mice by intraperitoneal (IP) injection of virus stock and then at graded times after infection performed radial arm water maze (RAWM) assays of learning and memory, euthanized cohorts, and collected tissues for measurement of viral DNA and RNA (Fig. 1a–c). A repeated measures ANOVA (RM-ANOVA) revealed a significant main effect of the period of time of EcoHIV infection (from virus inoculation to initiation of the RAWM testing) on the manifestation of symptomatic NCI in the RAWM test as reflected by the acuity of infected animals to find the submerged platform (F(4,115) = 29.887, p < 0.001 for retention trial (RT) errors and F(4,115) = 35.293, p < 0.001 for RT latency, other trials listed in Fig. 1 legend). Mice that began the RAWM 10 days after infection displayed slower spatial learning ability in finding the submerged platform in some learning trials compared to uninfected mice, but they became indistinguishable from uninfected mice during the RT, which measures working memory, one of the main cognitive deficits in PLWH^36^ (Fig. 1a–c). In contrast, when infection was allowed to proceed 25 or 55 days, mice were profoundly impaired in both spatial learning and working memory, suggesting progressive pattern of the disease. There was no significant main effect on disease in RM ANOVA in the control part of the test; all groups of mice tested were able to find and swim to a visible platform demonstrating that EcoHIV infection did not affect their motor, visual, and motivational competence (Fig. 1c). We conclude that induction of NCI by EcoHIV in mice is a dynamic process that can be assessed kinetically in RAWM. Infected mice show transient learning impairment within days of infection but develop a fully symptomatic disease including working memory deficit in about 3–4 weeks after infection. HIV DNA and RNA burdens in spleen reached an early peak, here measured 5 days after infection, and both then started to decline with HIV RNA barely detectable 20 days after infection (Fig. 1d–f). This result is consistent with our previously reported establishment of HIV transcriptional latency in mouse peripheral CD4^+^ T cells^15^ and with HIV transcriptional latency in these cells in PLWH^1^. Viral DNA in the brain was also detected within days of infection and was relatively stable over the first 3 weeks of the follow-up (Fig. 1g). PLWH and SIV-infected macaques show similar evidence of central nervous system infection within days of virus exposure^21,37^. Note that HIV brain burdens are significantly lower than peripheral virus burdens, consistent with observations in PLWH on effective CART who often have undetectable HIV RNA in cerebrospinal fluid^36^. In our experiments, EcoHIV RNA was not detectable by QPCR in total RNA isolated from brain tissue (not shown).

**Figure 2:**
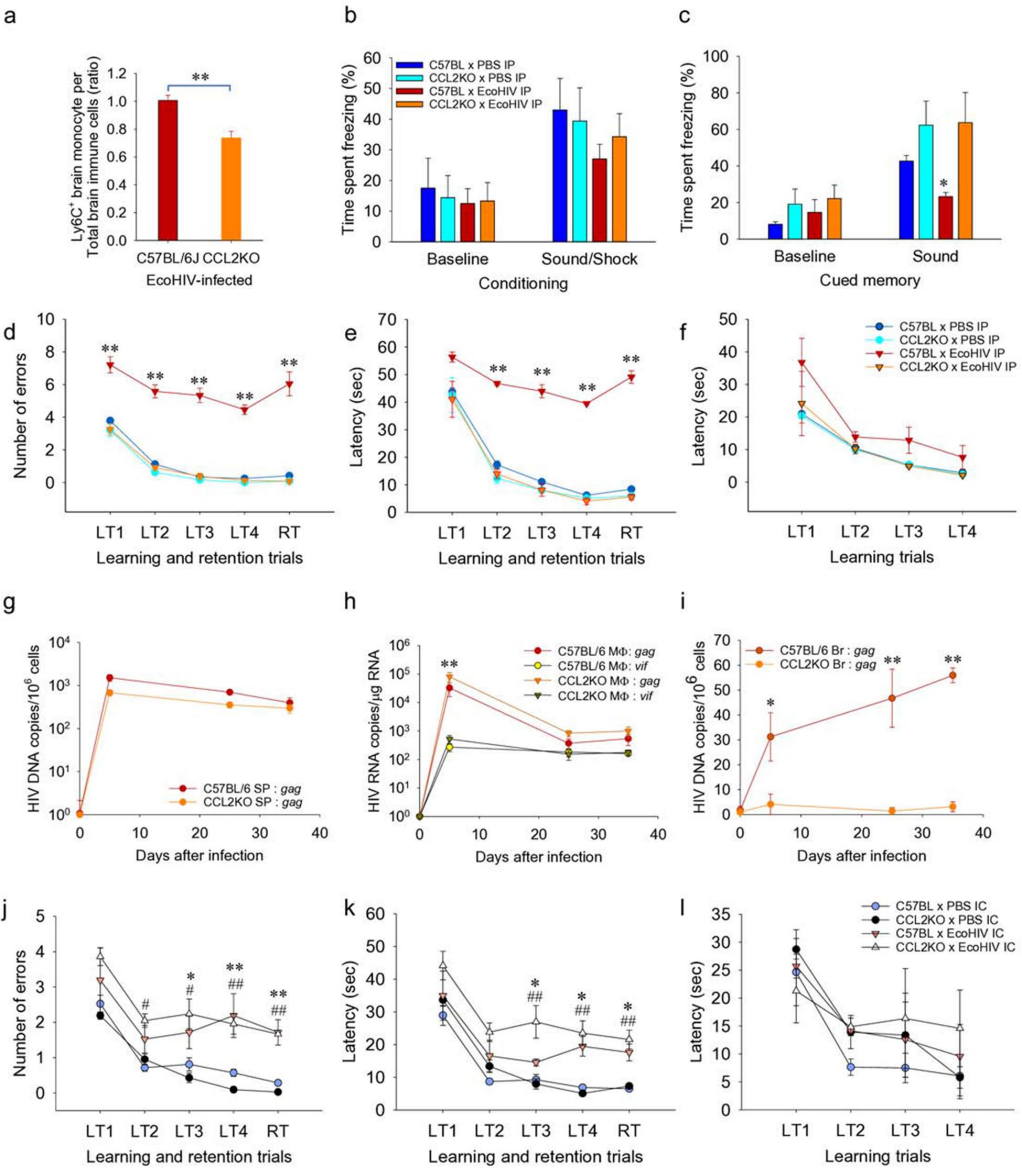
Migration of inflammatory monocytes to the brain and HIV-NCI development in EcoHIV infected CCL2KO compared to wildtype C57BL/6 mice. (**a**) 25 days after IP infection, brain immune cells were isolated and CD11b^+^LY6G^-^CD45^high^LY6C^+^ monocytes were measured by flow cytometry (n = 18/group). The ratio of CD11b^+^LY6G^-^CD45^high^LY6C^+^ cells to total brain immune cells is shown. **p < 0.01 C57BL/6 *vs.* CCL2KO, t-test. In another experiment, mixed-sex CCL2KO and C57BL/6 mice infected for 25 days (n = 6/condition, repeated once) were subjected to two behavioral tests to assess NCI. (**b**, **c**) results of the FC test showing mean percentages of time ± SEM spent freezing during (**a**) sound-shock conditioning on Day 1 and (**c**) auditory-cued recall on Day 2. *p < 0.05 C57BL/6xPBS *vs.* C57BL/6xEcoHIV, t-test. (**d**–**f**) results of the RAWM test. RM ANOVA: (**d**) F(3,68) = 26.275, p < 0.001/LT2, F(3,68) = 102.232, p < 0.001/LT3, F(3,68) = 46.078, p < 0.001/LT4; (**e**) F(3,68) = 28.144 p < 0.001/LT2, F(3,68) = 95.413, p < 0.001/LT3, F(3,68) = 97.241, p < 0.001/LT4. For RT values, see text. t-test, **p < 0.01 C57BL/6 × PBS *vs.* C57BL/6 × EcoHIV. (**g**–**i**) In another experiment, HIV burdens were measured at 5, 25, and 35 days after IP infection of CCL2KO and C57BL/6 mice (n = 12/group) by testing for HIV *gag* DNA in spleen (**g**), HIV *gag* and *vif* RNA in peritoneal macrophages (**h**), and HIV *gag* DNA in the brain (**i**). (**h**) t-test/day 5 p.i., **p < 0.01 C57BL/6 and CCL2KO macrophage *gag* RNA vs C57BL/6 and CCL2KO macrophage *vif* RNA; (**i**) t-test/days 5, 25, 35 p.i., C57BL/6 vs CCL2KO brain *gag* DNA, *p < 0.05 (day 5) and **p < 0.01 (day 25, 35). (**j**–**l**) mixed-sex C57BL/6 and CCL2KO mice were infected with EcoHIV by IC injection of virus and tested by RAWM 14 days p.i. (n = 6 per condition, repeated once). RM ANOVA: (**j**) F(3,80) = 4.035, p < 0.001/LT2, F(3,80) = 18.804, p < 0.001/LT3, F(3,80) = 45.484, p < 0.001/LT4; (**k**) F(3,80) = 1.776, p < 0.001/LT2, F(3,80) = 30.909, p < 0.001/LT3, F(3,80) = 26.538 , p < 0.001/LT4. For RT values, see text. t-test: *p < 0.05 and **p < 0.01 C57BL/6 × PBS *vs.* C57BL/6 × EcoHIV; ^#^p < 0.05 and ^#^p < 0.01: CCL2KO x PBS *vs.* CCL2KO × EcoHIV.

With the persistence of viral DNA in the brain and NCI manifestation, we inquired whether NCI development requires a minimum EcoHIV burden in the brain and performed virus dose response studies (Supplementary Fig. S1 online). RM ANOVA demonstrated a significant main effect of virus dose at infection on the manifestation of symptomatic NCI in the RAWM 25 days after infection (Fig. S1 in supplementary information online). The two lowest EcoHIV doses tested, 15 and 60 ng p24 per mouse, did not cause NCI in mice 25 days after infection and this correlated with very low HIV brain burdens, near the limit of detection of 10 copies viral DNA per 10^6^ cells. At EcoHIV infections from 0.25 μg p24 to 4 μg p24 per mouse, in the dose range used in the present experiments, mice were significantly impaired in RAWM and showed a dose-dependent increase in brain HIV DNA burdens (Fig. S1 online). These results suggest that NCI development in this animal model requires a minimum EcoHIV burden in the brain. Together, these results outline a time-period of infection required for establishment of neuropathogenic infection by EcoHIV in the brain and virus dose thresholds for cognitive impairment.

### Monocyte chemotaxis to CCL2 is a key contributor to EcoHIV NCI pathogenesis

Many previous studies implicate infected monocytes as vectors of HIV entry to the brain^37–40^. Indeed, we previously showed that leukocytes that infiltrate the brains of infected mice carry integrated EcoHIV DNA^15^. The likely signal for this migration is CCL2; it is the major monocyte chemokine^22^ and is highly induced by EcoHIV infection in mouse macrophages^16^ and in macrophages/microglia in the EcoHIV-or HIV-infected brain^10,41^. Direct injection of CCL2 into mouse hippocampus is a highly potent inducer of monocyte migration to the site^42^. We exploited a mouse strain with knockout of CCL2 (CCL2KO) and compared the course of its EcoHIV infection to that of wildtype C57BL/6 mice (Fig. 2). In the first series of experiments (Fig. 2a–j), CCL2KO and C57BL/6 mice were infected by IP EcoHIV inoculation to study NCI pathogenesis from infection to virus entry into the brain to behaviorally measurable NCI development 25 days after infection.

To assess directly the CCL2 contribution to monocyte migration to the brain, we isolated mononuclear cells from brains of both mouse strains 25 days after EcoHIV infection and performed flow cytometry to detect inflammatory monocytes defined as CD11b^+^Ly6G^neg^CD45^high^Ly6C^+^ as described^15,43^ (Fig. S2 in supplementary information on line). This critical cell type was significantly reduced in brains of EcoHIV-infected CCL2KO mice compared to infected wildtype mice (Fig. 2a). We next compared infected wildtype and CCL2KO mice for their performance in behavioral tests of learning and memory. Testing fear-associated learning and long-term associative memory functions in a fear conditioning test (Fig. 2b,c) revealed that infected wildtype mice were significantly impaired in these functions compared to uninfected mice, as we previously reported^10,15^. In contrast, infected CCL2KO mice resembled uninfected mice in these functions, suggesting that development of EcoHIV NCI requires a functional CCL2 gene (Fig. 2b,c). To confirm this result, the same groups of mice were subsequently tested for spatial learning and memory in RAWM (Fig. 2d–f). Infected C57BL/6 mice were again significantly impaired in this test while infected CCL2KO mice learned as well as uninfected mice (Fig. 2d, RM ANOVA for RT trial, F(3,68) = 72.845, p < 0.001; Fig. 2e, RM ANOVA for RT trial, F(3,68) = 97.241, p < 0.001). All mice tested were able to find and swim to a visible platform demonstrating that EcoHIV infection did not affect their motor, visual, and motivational competence (Fig. 2e). Thus, systemically EcoHIV infected CCL2KO mice appear refractory to HIV mediated behavioral deficits shown here and previously in wildtype mice^9,10,15^.

In another experiment, we tested whether presence or absence of NCI in the infected mouse strains correlated with peripheral and brain HIV burdens (Fig. 2g–i). Wildtype and CCL2KO mice were equally susceptible to EcoHIV infection of spleen cells and peritoneal macrophages over 5 weeks of observation (Fig. 2g,h, respectively). EcoHIV gag RNA was present at higher levels than vif RNA in macrophages early after infection, but the two viral RNA species reached similar stable levels on day 25 p.i. (Fig. 2h) consistent with chronic HIV infection in this animal model^15^. In contrast, EcoHIV DNA was at or below threshold of detection in the brain over 5 weeks of infection in CCL2KO mice compared to the detectable and increasing HIV brain DNA in infected wildtype mice (Fig. 2i). The EcoHIV dose response studies in mice shown in Supplementary Fig. S1 online suggest that there is a threshold of HIV brain burden in mice required for manifestation of symptomatic NCI in RAWM tests. The results shown in Fig. 2i, combined with behavioral data in Fig. 2b–f, indicate that HIV DNA brain burdens in infected wildtype mice reach this threshold and in CCL2KO mice do not, despite efficient infection of spleen T cells and peritoneal macrophages in both mouse strains mice. Recent studies suggest that CCL2 preferentially mediates transmigration of HIV infected mature monocytes across a model of the blood–brain-barrier in culture^31,44^. If CCL2 also preferentially attracts HIV-infected monocytes to the brain in vivo, the results shown in Fig. 2a–f suggest that the observed low HIV brain burdens and absence of NCI in EcoHIV-infected CCL2KO mice can be attributed to the loss of CCL2 driven EcoHIV-infected monocyte trafficking to the brain.

We next inquired whether CCL2 plays a similar role in established neurocognitive disease in EcoHIV infected mice. Direct EcoHIV infection of mouse brain by intracranial (IC) injection allows efficient infection of microglial cells and causes symptomatic neurocognitive disease within 2 weeks of virus brain inoculation^9^. In the present context, we reasoned that IC infection of CCL2KO mice may override the reduction in neuropathogenic EcoHIV entry observed during IP infection of these mice (Fig. 2i). We infected the brains of wildtype and CCL2KO mice by stereotaxic IC inoculation into the striatum region^7,9,10^ and 2 weeks later measured the learning and memory functions of infected and control mice in RAWM (Fig. 2j–l). The results in Fig. 2j,k show that IC infection significantly impaired learning/memory in both wildtype and CCL2KO mice compared to uninfected mice (Fig. 2j, RM ANOVA for RT trial, F(3,80) = 31.145, p < 0.001; Fig. 2k, RM ANOVA for RT trial, F(3,80) = 26.572, p < 0.001), but affected neither vision, motor function, nor motivation of any group of mice to seek the visible platform (Fig. 2l). Taken together, our results with CCL2KO mice strongly indicate a requirement for CCL2 for HIV entry into the brain and establishment of symptomatic NCI in this animal model. Other monocyte chemoattractants could participate in these processes^45^ but they are insufficient for promoting HIV-NCI in systemically infected CCL2KO mice. On the other hand, the ability of brain resident cells in IC infected mice to cause NCI in the absence of the chemokine CCL2 in CCL2KO mice (Fig. 2j–k) suggests that CCL2 plays different roles during initiation of neurocognitive disease and after brain infection has been established.

### Knockout of CCL2 or its receptor CCR2 impairs EcoHIV infection of macrophages in culture

**Figure 3:**
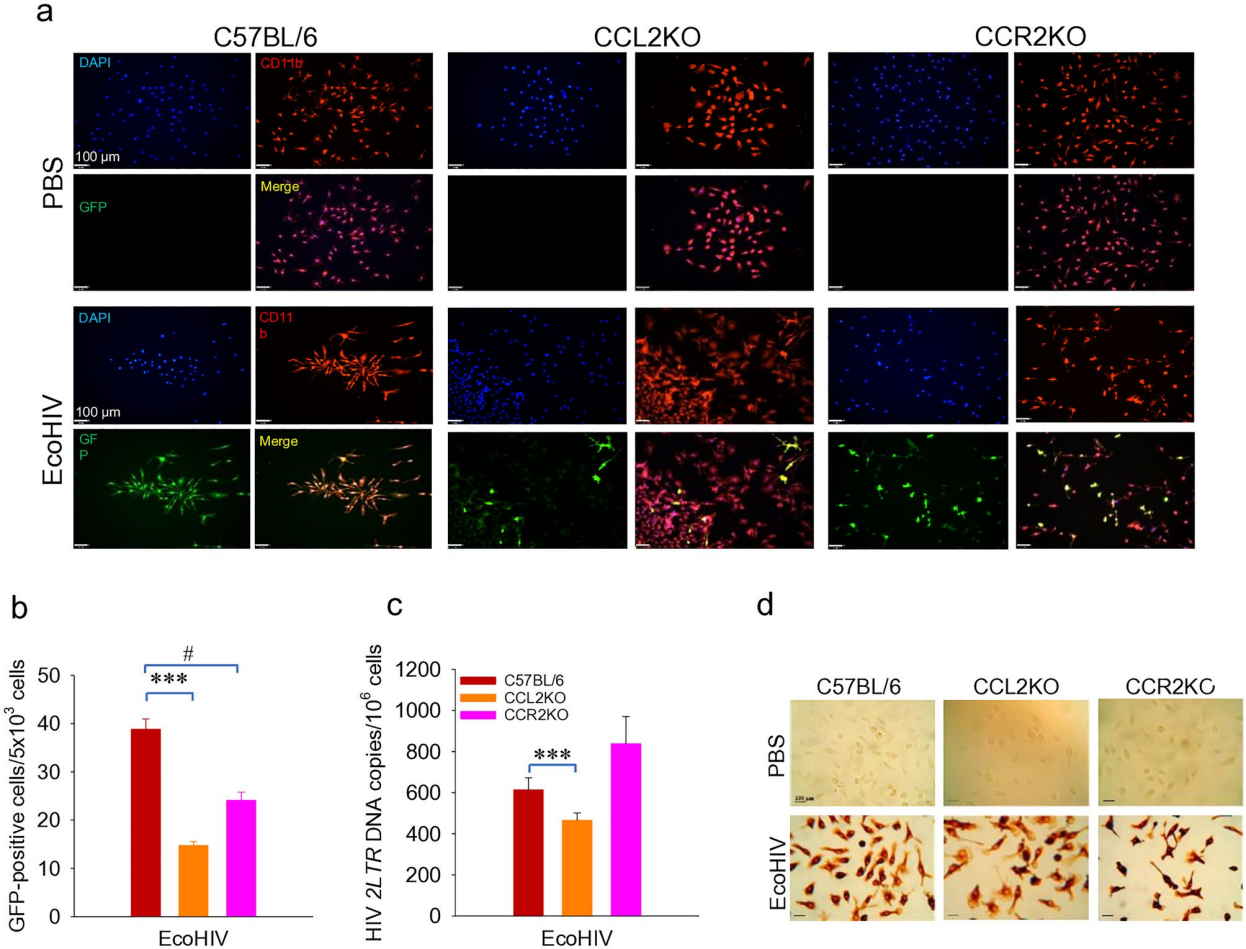
EcoHIV infection of BMM from C57BL/6, CCL2KO, and CCR2KO mice. See Methods for details. (**a**) Detection of EcoHIV-EGFP infected cells by fluorescence microscopy. EcoHIV-EGFP, green; CD11b, red; DAPI, blue; Merge, yellow. Scale bar = 100 μm. (**b**) Measurement of EcoHIV-EGFP expression by microscopy and image analysis. ***p < 0.001 C57BL/6 *vs*. CCL2KO, ^#^p < 0.05 C57BL/6 *vs*. CCR2KO. (**c**) Measurement of 2-LTR DNA by QPCR. ***p < 0.001 C57BL/6 *vs*. CCL2KO. (**d**) Immunohistochemistry staining for HIV p24.

Previous studies indicate that CCL2 promotes HIV infection in culture^23,24^ but our results in Fig. 2g,h show that in vivo, EcoHIV infected wildtype and CCL2KO mice have similar viral burdens in spleen and macrophages, indicating that these mouse strains are equally susceptible to peripheral HIV infection. We then assessed the role of CCL2 and its major receptor, CCR2, in susceptibility of macrophage to EcoHIV in culture. We employed 1-week infection in tissue culture of fully differentiated bone marrow macrophages (BMM) from mouse strains C57BL/6, CCL2KO, and CCR2KO (Fig. 3). We performed fluorescence microscopy and image analysis to count cells expressing enhanced green fluorescent protein (EGFP) from EcoHIV carrying the marker gene downstream of *nef*^15^ and co-stained cells for macrophage marker CD11b (red) and cellular DNA (blue) (Fig. 3a). Knockout of CCL2 or CCR2 significantly impaired BMM infection measured by EGFP expression (Fig. 3b). We also conducted QPCR to measure newly synthesized, 2-LTR circular HIV DNA and found a significant reduction in this viral DNA form in CCL2KO but not CCR2KO macrophages compared to wildtype BMM (Fig. 3c), suggesting the presence of an additional restriction to HIV expression in CCR2KO macrophages compared to CCL2KO macrophages after DNA synthesis. Finally, we repeated the experiment and using immunohistochemistry, we counted cells expressing HIV p24 and found reduced virus expression in both CCL2 and CCR2KO cells (Fig. 3d). Since macrophages from infected C57BL/6 and CCL2KO mice are infected to similar levels, our results suggest that in vivo susceptibility to or expression of EcoHIV is affected by potentially more complex interactions among diverse cell types and their production of chemokines and cytokines in a living organism than can be reproduced in culture.

### The prophylactic pharmacological block of CCL2 synthesis also prevents development of HIV-NCI

**Figure 4:**
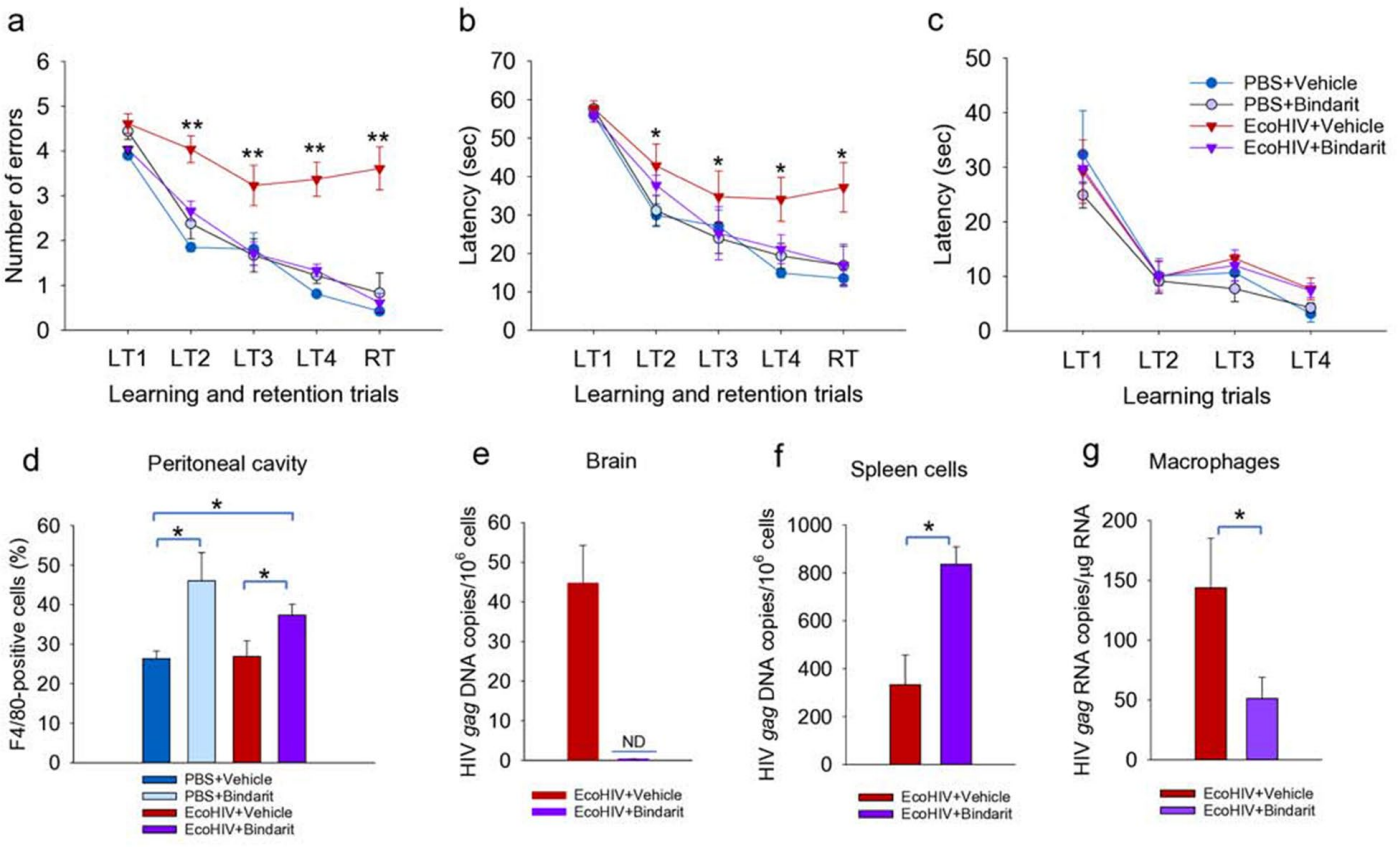
Pharmacological inhibition of CCL2 driven monocyte/macrophage migration in mice prevented EcoHIV induced memory defects. Male 6–8-week-old C57BL/6 mice were treated with the CCL2 synthesis inhibitor bindarit or placebo prior to and during EcoHIV infection. Learning and memory were measured using RAWM by counting (**a**) errors and (**b**) time to find the hidden platform or (**c**) time to find visible platform. RM ANOVA: (**a**) F(3,80) = 15.114, p < 0.001/LT2, F(3,80) = 21.837, p < 0.001/LT3, F(3,80) = 28.48, p < 0.001/LT4; (**b**) F(3,80) = 3.967, p < 0.001/LT2, F(3,80) = 7.463, p < 0.001/LT3, F(3,80) = 29.007, p < 0.001/LT4. For RT values, see text. Pairwise t-test, *p < 0.05 **p < 0.01 PBS *vs.* EcoHIV. (**d**) F4/80 positive macrophages were measured in the peritoneal cavity by flow cytometry. *p < 0.05 vehicle versus bindarit. (**e**, **f**) HIV *gag* DNA was measured in (**e**) brain and (**f**) spleen, or (**g**) HIV *gag* RNA was measured in peritoneal macrophages *p < 0.05 EcoHIV + vehicle vs. EcoHIV + bindarit. ND = not detected.

To test the effects of CCL2 upon initiation of HIV brain disease by an alternate approach, we employed the compound bindarit that inhibits transcription of CCL2 through interference with NFκB activation at the CCL2 promoter^35^. In mice, bindarit blocks expression of CCL2 RNA and protein and prevents inflammatory monocyte entry into the brain^46^ and it also prevents pathological microglia activation during impaired neonatal brain development^47^. C57BL/6 mice were pretreated with bindarit 1 day before EcoHIV infection by IP injection and then treated daily through RAWM testing starting on Day 25 until euthanasia on Day 32 (Fig. 4). Consistent with results shown in Figs. 1 and 2, EcoHIV-infected untreated mice showed significant learning and memory impairment in RAWM compared to uninfected control mice (Fig. 4a,b). However, RM ANOVA in combination with post hoc and t-tests demonstrated that mice treated with bindarit in an experimental prophylaxis design learned as well as uninfected mice (Fig. 4a, RM ANOVA for RT trial, F(3,80) = 43.312, p < 0.001; Fig. 4b, RM ANOVA for RT trial, F(3,80) = 48.104, p < 0.001), indicating a block to NCI pathogenesis by bindarit prophylaxis (Fig. 4a,b). Bindarit administration had no effect upon performance of uninfected mice in RAWM and in no case motor, visual, or motivational abilities of mice were affected (Fig. 4c). To evaluate the effects of bindarit upon monocyte/macrophage trafficking we measured cells in the peritoneal cavity staining for the mouse myeloid marker F4/80. Bindarit increased the number of F4/80 positive peritoneal cells in both infected and uninfected mice indicating some restriction in exit from this compartment (Fig. 4d). Measurement of virus burden revealed that after bindarit treatment, EcoHIV was undetectable in the brain, significantly increased in spleen and significantly reduced in peritoneal macrophages (Fig. 4e–g). The virological findings from Fig. 4e are consistent with those from systemically infected CCL2KO mice (Fig. 2b–f) and indicate that CCL2 is required for efficient monocyte migration and HIV entry into the brain, functions essential for the development of HIV NCI. The effect of bindarit treatment on HIV burdens in spleen and macrophages (Fig. 4f,g, respectively), on the other hand, may reflect the interference of the drug with NFκB and it requires further study.

### Block of CCL2 synthesis does not affect established EcoHIV brain infection or disease

**Figure 5:**
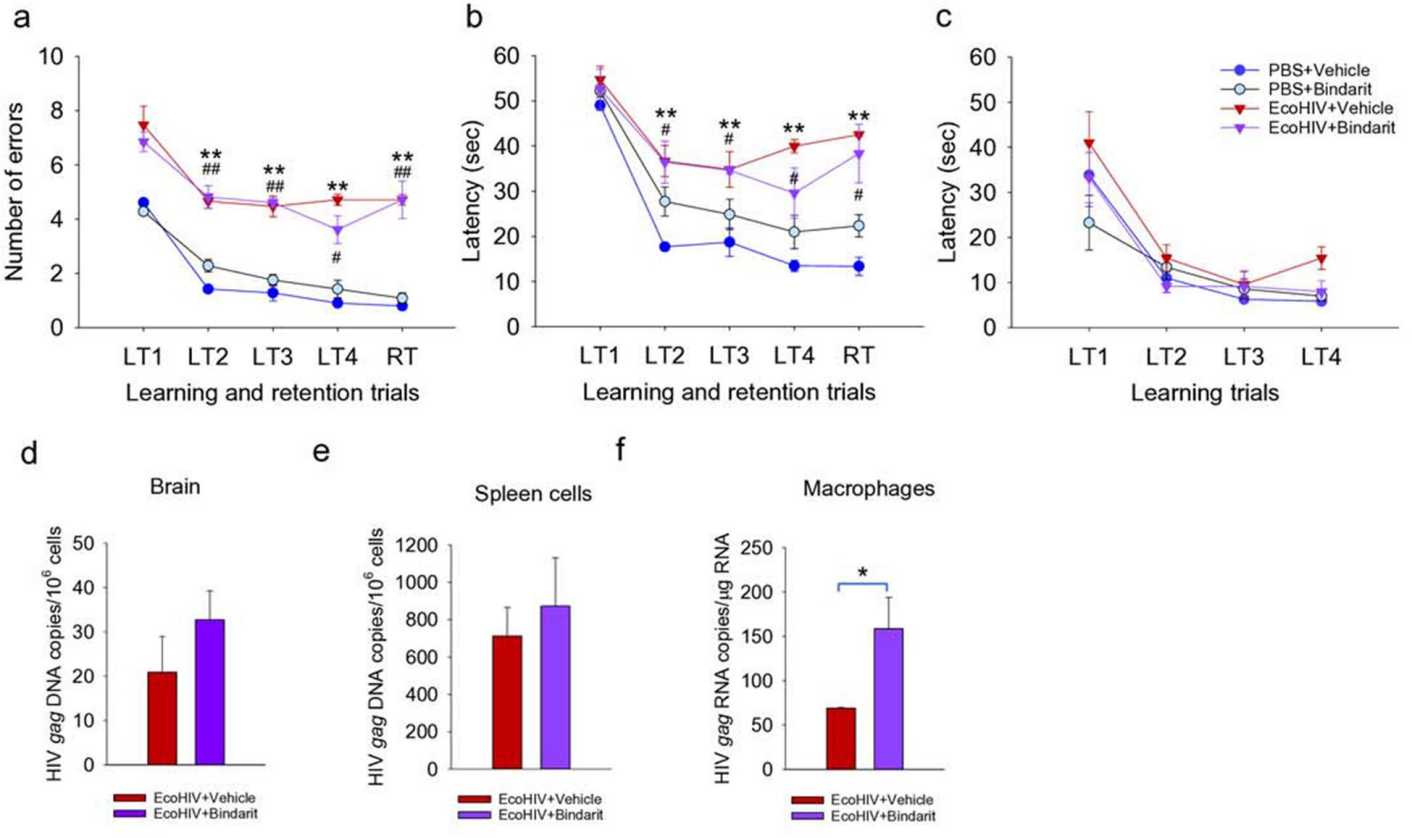
Inhibition of CCL2 driven monocyte/macrophage migration in chronically infected mice does not affect established EcoHIV induced learning/memory defects. Four weeks after EcoHIV infection of male 6–8-week-old C57BL/6 mice, the animals were treated daily with bindarit for 2 weeks prior to behavioral test and euthanasia. RAWM was conducted by measurement of (**a**) errors and (**b**) time to find the hidden platform or (**c**) time to find the visible platform; n = 17 per group. RM ANOVA: (**a**) F(3,80) = 41.265, p < 0.001/LT2, F(3,80) = 36.379, p < 0.001/LT3, F(3,80) = 47.972, p < 0.001/LT4; (**b**) F(3,80) = 34.142, p < 0.001/LT2, F(3,80) = 25.528, p < 0.001/LT3, F(3,80) = 37.372, p < 0.001/LT4; For RT values, see text. Pairwise t-test, **p < 0.01 PBS + vehicle and PBS + bindarit vs. EcoHIV + vehicle, ^#^p < 0.05 and ^##^p < 0.01 PBS + bindarit versus EcoHIV + bindarit. (**d**, **e**) HIV *gag* DNA was measured in (**d**) brain and (**e**) spleen cells, or (**f**) HIV *gag* RNA was measured in peritoneal macrophages. *p < 0.05 EcoHIV + vehicle vs. EcoHIV + bindarit.

The ability to conditionally eliminate CCL2 with bindarit^35^ permits investigation of the role of CCL2 in NCI and the persistence of EcoHIV in the brain of systemically infected mice. Mice were infected by IP injection and allowed 4 weeks for development of symptomatic NCI in accordance with NCI kinetics studies in Fig. 1; then bindarit was administered daily for 1 week prior to and during the RAWM for a total of 2 weeks of drug treatment. Mice were then euthanized for tissue collection and measurement of virus burdens (Fig. 5). As shown in Fig. 5a,b by a combination of RM ANOVA with Bonferroni post hoc test and t-test, mice with and without bindarit treatment were similarly impaired in learning and memory in RAWM tests (Fig. 5a, RM ANOVA for RT trial, F(3,80) = 59.396, p < 0.001; Fig. 5b, RM ANOVA for RT trial, F(3,80) = 60,789, p < 0.001), suggesting that EcoHIV-infected mice continued to manifest learning and memory defects regardless of bindarit treatment. Neither vision, motor function, nor motivation of any group of mice to seek the visible platform were affected in RM ANOVA and post hoc analyses (Fig. 5c). Testing HIV *gag* DNA burdens in the brain revealed no significant effect of bindarit (Fig. 5d), consistent with the observations in behavioral tests (Fig. 5a,b). However, virus burden in macrophages was significantly increased (Fig. 5f), likely due to inhibition of exit from the peritoneal cavity as shown in Fig. 4g. The prevention of EcoHIV brain infection when bindarit was administered prior to infection (Fig. 4) and its inability to affect the maintenance of brain virus and disease when begun at the same dose 4 weeks after infection (Fig. 5) is consistent with the development of HAND in PLWH despite suppressive ART^36,48,49^ and suggests that interventions to treat HAND must act in the brain.

## Discussion

We report that through its promotion of infected monocyte migration, CCL2 is required for HIV brain entry and development of HIV NCI during systemic EcoHIV infection of mice. However, once established, HIV NCI can persist in mice in the absence of CCL2. One clinical implication of these findings is that mitigation of NCI in PLWH may entail purging pathogenic HIV reservoirs in the brain regardless of the status of peripheral virus. At present, neither CART nor the partially restored anti-HIV immune responses in PLWH can handle this task^1,2,50^.

The kinetics of EcoHIV replication after IP inoculation (Fig. 1) indicate that following the initial peak, viral DNA burdens in spleen cells decline moderately consistent with results we previously reported^15^. The decline in viral DNA in the spleen may reflect cytopathic effects of anti-viral CD8^+^ T cells that we found induced by EcoHIV infection in mice^17^. In contrast, viral DNA burden is roughly stable in the brain, possibly because of the relative resistance of infected myeloid cells to CD8^+^ T cell cytotoxicity^51^. The time-course of NCI development indicates a progressive disease. Mice infected for up to 10 days show mild and transient spatial learning impairment and no working memory deficits, but mice infected for 25 days and longer are highly impaired in these cognitive domains as well as fear responses, suggesting a broad injury to multiple brain regions involved in these functions and consistent with other studies in this model^8–11,13^. We previously reported that NCI in EcoHIV-infected mice persists for at least 14 weeks^5,10,15^. Together, these results suggest that NCI pathogenesis in EcoHIV-infected mice occurs in two phases, an early, preclinical phase during the first 1–2 weeks after systemic infection followed by a transition to a clinical/behaviorally symptomatic phase from 3 to 4 weeks after infection (Figs. 1, 2). Such disease pattern is typical in human diseases including HAND^36^ where it may take several years from HIV infection and CART initiation until symptomatic NCI manifestations^48^. Understanding the mechanisms involved in the pre-symptomatic-to-symptomatic NCI transition could be key to controlling disease progression.

The data shown here suggest that the pre-symptomatic-to-symptomatic NCI transition in EcoHIV-infected mice may require passing the threshold of HIV brain infection levels needed for symptomatic NCI manifestation. CCL2 preferentially promotes HIV-infected monocyte migration in blood–brain-barrier models^31,44^, and its levels in the CNS correlate with cognitive dysfunction in HIV infection in PLWH^34^. During HIV infection of human beings, viral RNA was detected in 15/18 CSF samples tested with elevated CCL2 found in 5 viral positive samples^21^. Later in infection, CCL2 levels in CSF but not in plasma correlate with increased CSF HIV RNA and the development of HIV encephalitis^52^. Elevated CCL2 was also found in brains and CSF of patients with HIV dementia^25^ and CCL2 has been shown to promote HIV infection in monocytoid cells in culture^28,29^ and in vivo^52,53^.

To exclude CCL2 activity to allow detection of the function of any other factors^54^ to promote infected monocyte migration and infection of the brain, we used existing mouse knockouts of CCL2 and found that they fail to develop HIV-NCI during systemic EcoHIV infection but succumb to the disease when the virus is injected into the brain (Fig. 2). Brain inflammation and induction of CCL2 are prominent after IC EcoHIV infection of wildtype mice^7,9^. However, NCI observed here after IC infection of CCL2KO mice (Fig. 2j–l) manifests in the absence of a functional CCL2 gene. This suggests that once HIV brain infection and HIV-NCI are established, the CCL2-mediated^22,41,54^ influx of monocytes to the brain is not required. These results indicate that EcoHIV must be present in the brain to cause disease, either after direct injection shown here or by CCL2 driven entry of infected monocytes from the periphery into the brain, the latter route previously indicated^37–40^. These findings were futher corroborated here by blocking EcoHIV entry into the brain during systemic virus infection of wildtype mice in the presence of bindarit (Fig. 4). Bindarit treatment prior to EcoHIV infection of mice impeded macrophage exit from the peritoneal cavity, increased EcoHIV replication in spleen, fully blocked EcoHIV infection of the brain, reduced infection of peritoneal macrophages, and prevented development of NCI (Fig. 4). Consistent with these findings, we reported previously that brain infiltrating leukocytes in EcoHIV-infected mice carry integrated HIV DNA^15^. A collaborative group including some of the current authors reported that buprenorphine, an opiate therapeutic^55^, administered prior to EcoHIV infection of mice, reduces inflammatory monocyte entry to the brain and prevents NCI^43^. That study and our data shown here (Figs. 2 and 4) strongly support the necessity of infected monocyte migration to the brain during primary EcoHIV infection for NCI pathogenesis. Importantly, the two studies confirm the centrality of CCL2 signaling in the HIV-infected monocyte migration process^31,44^. In the brain, the absence of disease and near absence of EcoHIV DNA in CCL2KO mice after IP infection (Fig. 2) also indicate that other chemotactic factors^45,53^ do not compensate for the lack of CCL2 in seeding HIV in the brain above the viral burden threshold required for NCI pathogenesis. These findings, consistent with previous reports^30^, indicate that CCL2 is the major chemokine responsible for initial HIV entry into the brain through monocyte migration.

During HIV infection of macrophages in culture CCL2 levels or signaling and virus replication are positively correlated. Exogenous CCL2 increases HIV replication^29^ and CCL2 neutralization decreases HIV replication^28^. We found that knockout of either CCL2 or CCR2 slightly reduced EcoHIV infection of BMM in culture depending upon the assay. CCR2 appears to be the major receptor driving HIV-infected monocyte migration to CCL2 across a blood–brain-barrier^45^. However, CCL2KO did not affect infection of macrophage in mice suggesting that the cellular and soluble factor environment for EcoHIV infection in vivo is less dependent upon CCL2 than in macrophage culture.

Administration of bindarit during chronic virus infection offered an additional approach to inquire whether CCL2 functions are required to maintain NCI. While the CCL2 synthesis inhibitor was highly effective in preventing NCI (Fig. 4), we found that it failed to affect NCI in mice with established disease, also it failed to affect virus burden in spleen or the brain (Fig. 5). The apparent increase of virus burden in macrophages in bindarit treated mice may arise from its inhibition of macrophage exit from the peritoneal cavity (Fig. 4). Thus, two avenues that test the need for continued monocyte migration to the brain to maintain NCI, either by IC infection of CCL2KO mice which lack a functional CCL2 gene (Fig. 2) or bindarit treatment in chronic infection of wildtype mice (Fig. 5), suggest that cells resident in the brain, likely perivascular macrophages and microglia, maintain EcoHIV infection and its pathogenic effects upon cognitive function regardless of potential loss of CCL2 driven monocyte migration to the brain. A collaborative group including some of the coauthors of the present work recently reported that NCI is reversed by treatment of EcoHIV-infected mice with buprenorphine^56^. Buprenorphine treatment also reduced HIV burdens and inflammatory monocyte levels in the brain^56^, suggesting that continuing migration of HIV-infected monocytes to the brain is required to maintain HIV-NCI. However, since buprenorphine also affects various aspects of behavior^57,58^, additional mechanisms may apply. The ability to reverse NCI is consistent with the state of brain neuropathology in PLWH with mild NCI and in our model of mice with EcoHIV NCI. Unlike HIV dementia, both are characterized primarily by broad synaptodendritic injury and brain metabolic abnormalities but not extensive neuronal death^9–11,36,59^, suggesting that injured neurons could regain some functions. Supporting this concept, longitudinal neuropsychological (NP) tests in large PLWH cohorts showed that their neurocognitive functions can fluctuate over time, depending on the individual NP criteria tested^60^, including improvements in some NP metrics in subpopulations of patients^60–62^. We and collaborators reported that treatment of EcoHIV infected, cognitively impaired mice with intranasal insulin reverses synaptodendritic injury and NCI^10^, possibly by increasing brain energy metabolism^63^. The virus brain burdens were not stably altered in this study despite continuing insulin therapy and NCI improvement^10^ suggesting that that some interventions can mitigate NCI without reducing brain HIV burdens. NCI reversal was also observed after treatment of EcoHIV-infected mice with the glutaminase antagonist JHU083^13^, potentially through restoration of physiological glutamate levels in the brain^12^, or a novel neutral sphingomyelinase inhibitor PDDC, the latter possibly through normalization of elevated ceramide and extracellular vesicles production found in EcoHIV infected mice and PLWH^64^. The NCI in the latter study was measured for the first time by the extent of psychiatric-like deficits^64^, adding to the range of neurocognitive deficits in EcoHIV infected mice resembling these seen in PLWH^36^. Overall, these studies indicate that addressing dysregulated neurobiological functions in the brain at several levels can ameliorate HIV behavioral defects without eliminating brain HIV, suggesting the possibility of a functional cure of NCI.

Of particular relevance to the work presented here are reports that CCL2 overexpression by a promoter variant increases the risk of HIV associated dementia in human beings^33^ and that the extent of this overexpression correlates with the extent of cognitive impairment observed^34^. Although direct evidence of the impact of CCL2 overexpression upon macrophage entry into the brain in PLWH is not available, a similar phenomenon of increased macrophage entry into tissue correlated with the CCL2 promoter variant during cell migration to the kidney during SLE nephritis^33^. The results presented here place the role of CCL2 in NCI as the signal to HIV-infected monocytes to enter the brain where infection and disease ensue. Once the disease is established, we find that continued NCI during EcoHIV infection of mice persists in the absence of CCL2 and further monocyte migration or response to CCL2. Results presented here coupled with clinical findings that HIV enters the human brain roughly within a month of infection^21^ and that HIV suppressive CART, generally administered later, fails to block HAND^1–3^ suggest that effective reversal of HAND should target neuropathogenic events within the brain itself.

## Materials and methods

### Mice, cells, and tissue culture

The protocols for all experiments involving animal use were executed with the approval of the Icahn School of Medicine at Mount Sinai Animal Care and Use Committee under the protocol IACUC-2014-0124 (DJV), renewed on August 11, 2022. All experiments using mice were designed and conducted with the approval of Icahn School of Medicine at Mount Sinai Animal Care and Use Committee and according to all relevant guidelines and regulations including all relevant ARRIVE guidelines including reporting. All mice were purchased from Jackson Laboratory including C57BL/6 (stock# 000664), B6.129S4-CCL2 (CCL2 KO mice, strain stock# 004434), and B6.129S4-Ccr2^tm1Ifc^/J (CCR2 KO) mice, strain stock# 004999). Cohorts of 6–8-week-old male C57BL/6 mice and mixed-sex cohorts of CCL2KO mice at an approximate 1:1 ratio were used. After virus infection, mice were euthanized under methods approved by the Icahn School of Medicine at Mount Sinai Animal Care and Use Committee and peritoneal macrophages, spleens, and brain tissues were removed and prepared for measurement of HIV burden, cellular gene expression and microscopy as described^5,9,15^. For BMM culture, 8–10 weeks old mice were sacrificed by carbon dioxide asphyxiation. Marrow was harvested from hind legs, erythrocytes were lysed using ACK lysing buffer (Lonza, Walkersville, MD) for 5 min at room temperature, and nucleated cells were cultured in RPMI1640 with 10% horse serum, 5% fetal bovine serum, 100 units/ml penicillin/streptomycin (ThermoFisher Scientific, Waltham, MA), and 20% L929 conditioned medium^65^. For infection, cells were cultured in complete culture medium without antibiotics 24 h before infection. Cells were then infected with virus at 1 pg p24 per cell in culture medium without serum and antibiotics for 3 h then replaced the medium to complete culture medium and incubated in 5% CO_2_ at 37 °C for 7 days.

### Virus construction and preparation

EcoHIV/NDK-V5 and EcoHIV/NDK-EGFP were constructed as described^10,15^ respectively and virus stocks were prepared as described^5^.

### Preparation of bindarit and treatment

Bindarit, 2-[(1-benzylindazol-3-yl)methoxy]-2-methylpropanoic acid (MW 324.37), was synthesized by Biorbyt (Biorbyt Limited, UK) and was prepared as a suspension in 0.5% methylcellulose (MTC) at a concentration of 20 mg/ml as previously described^66^. Animals were injected IP with bindarit, suspended in 0.5% MTC aqueous solution, at the dose of 100 mg/kg daily^67^ either for prevention from 1 day before EcoHIV infection until euthanasia or for treatment starting 4 weeks after infection until euthanasia.

### Virus or vehicle injection

IP injections were in a volume of 0.5 ml. Infection was conducted at a dose of 2.0 × 10^6^ pg p24 unless otherwise stated. Intracranial infection was conducted as described^7,9,17^.

### Behavioral tests

Learning and memory were evaluated in groups of 8 to 10 mice using the RAWM and auditory-cued fear conditioning FC tests as the authors have described previously^7,9,15^ Briefly, RAWM testing consisted of four training trials (LT), followed by a retention trial (RT) administered after a 30-min rest, repeated daily until control mice reached asymptotic performance of one error or fewer on trials LT4 and RT, usually between 3 and 5 consecutive days of testing. Errors for the last 3 days of testing were averaged for statistical analysis. The hidden platform tests were followed by measuring the latency for finding a visible platform as a control for possible effects of treatment on animal vision, motivation, and motor ability. FC testing was conducted using an SDI Freeze Monitor (San Diego Instruments, San Diego, CA). Conditioning sessions included three consecutive pairings of 10-Hz sound signals and 0.7-mA electric shock signals; cued associative fear memory was measured the following day in a novel context by presenting sound signal alone. Results are shown as the mean total percentage of time spent freezing pre- and post-cue on both the conditioning and the cued memory days.

### Fluorescence microscopy and immunohistochemistry

For fluorescence microscopy, cells were spotted on slides and fixed with 4% paraformaldehyde in PBS, permeabilized with 0.2% Triton X-100 (Sigma-Aldrich, Burlington, MA) at 4ºC for 10 min, treated with 5% normal donkey serum (Jackson, USA) in PBS for 1 h, and washed with PBS. Cells were incubated with anti-EGFP (1:500, ThermoFisher Scientific), anti-CD11b (1:100, BD Pharmingen, San Diego, CA), and anti-p24 (1:50, H12 clone, NIAID U.S.A.). For secondary antibody binding, anti-chicken Alexa 488 (1:200; ThermoFisher Scientific) and anti-mouse Alexa 555 (1:100; ThermoFisher Scientific) were suspended in PBS and incubated for 1 h and washed with PBS three times then were conjugated, DAPI (ThermoFisher Scientific) staining was used as a nuclear marker and observed with fluorescence microscopy (Zeiss, Inwood, NY). Cells positive for specific markers were counted under fluorescence microscope for statistical analysis (Volocity, Quorum Technologies, Inc.). Briefly, 5 × 10^3^ DAPI positive cells were counted for EGFP expression and presented as percent positive. For p24 staining Catalyzed Signal Amplification System (Dako, Santa Clara, CA) was used with H12 Ab clone 183-H12-5c, then observed with inverted microscopy (Nikon, Garden City, NY). Experiments were repeated at least three times.

### Quantitative real-time RT-PCR (QPCR)

For QPCR, RNA and DNA were isolated from cells and tissues as described^15^ and amplification was conducted as described^15,68^.

### Flow cytometry for peritoneal macrophages and brain-infiltrating monocytes

With nine mice per experimental group, peritoneal cells were collected by washing the peritoneal cavity with 10 ml PBS containing 0.3 M sucrose. Cells were stained for mouse F4/80 macrophage cell surface marker using mAb clone REA126, FITC labeled [Milteny Biotech, Auburn, CA, cat. no. 130-102-988] diluted 1:10 in PBS containing 1% FBS and 0.05% sodium azide (0.15 μg antibody used per sample). Cells were analyzed using Accuri C6 flow cytometer^69,70^. The brain immune cells from mouse were isolated with enzymatic digestion followed by Percoll density gradient centrifugation as previously described^15,71^. The marker combination of CD11b^+^LY6G^-^CD45^high^LY6C^+^ was used for identifying brain-infiltrating inflammatory monocytes^15,43,72^. Before staining cells Fc-receptors were blocked with anti-mouse CD16/32 (eBioscience, ThermoFisher Scientific, Cat# 2023-07-0) following the manufacturer’s instructions. Cells were stained in PBS with 3% FBS with the following antibodies: CD11b-PE-Cy7 (Biolegend, San Diego, CA, Cat# 101215), CD45-FITC (Biolegend, Cat# 103107), Ly6C-PE (Biolegend, Cat# 128007), and Ly6G-PerCP-Cy5.5 (Becton Dickinson, Franklin Lakes, NJ, Cat# 560602). All antibodies were used according to manufacturer instructions. Fluorescence minus one sample was used for determining the background staining for each antibody. Flow cytometry data were acquired on Attune NxT Flow Cytometer (ThermoFisher Scientific) or LSRII flow cytometer (BD Biosciences, Franklin Lakes, N). Flow data analysis was performed using FlowJo (Three Star) software. Two separate experiments were run with n = 9 mice per group each time.

### Rigor and reproducibility

Each experiment described here was conducted at least twice with similar findings.

### Statistical analysis

Statistical analysis was conducted with SigmaPlot 14.0 software. RAWM were analyzed for within-subject variability using repeated measures one-way analysis of variance (RM ANOVA) with power of performed test with alpha < 0.05 and Bonferroni post hoc pairwise comparison with significance defined as p < 0.05. The independent variable was defined as experimental conditions including treatment, time after infection, etc. The dependent RM variable was defined as errors/latency measured over time (for the last 3 days of RAWM testing) for the indicated Trial. Bonferroni ANOVA pairwise comparisons were extended with unpaired Student’s t-test with p values shown by asterisks or hashtags in Figures. RM ANOVA results for RT are listed in the text, F values for other trials are listed in Figure legends. Differences in HIV burdens and other parameters between controls, infected, or treated mice were tested by one-way ANOVA with Holm-Sidak post hoc analysis and t- test, t-test significance symbols are shown in Figures. Changes in cellular gene expression in brain tissues of infected mice were first normalized to respective uninfected controls, with comparisons being made among each group. t-test p value representations are: *p < 0.05; **p < 0.01; ***p < 0.001.

## Supplementary Information

Supplementary Figures.

## Data Availability

The datasets used and/or analyzed during the current study will be available from the corresponding author on a reasonable request.
