# Supplementary material for: CCL2 is required for initiation but not persistence of HIV infection mediated neurocognitive disease in mice
Source: Sci Rep. 2023 Apr 21;13:6577. doi: 10.1038/s41598-023-33491-7 (PMC10121554; doi:10.1038/s41598-023-33491-7)
Supplement: Supplementary file 1 — Supplementary Figures. [file 41598_2023_33491_MOESM1_ESM.pdf]

CCL2 is required for initiation but not persistence of HIV infection mediated neurocognitive disease in mice.

Boe-Hyun Kim[1], Eran Hadas[1], Jennifer Kelschenbach[1], Wei Chao[1], Chao-Jiang Gu[1,2]
Mary Jane Potash[1], and David J. Volsky[1]*

[1]Division of Infectious Diseases, Department of Medicine, Icahn School of Medicine at Mount Sinai, New York, NY 10029

[2]Current address: College of Life and Health Sciences, Institute of Biology and Medicine, Wuhan University of Science and Technology, Wuhan, Hubei, China

*Corresponding author: Division of Infectious Diseases, Department of Medicine, Icahn School of Medicine at Mount Sinai, 1468 Madison Avenue, New York, NY 10029. Email: david.volsky@mssm.edu

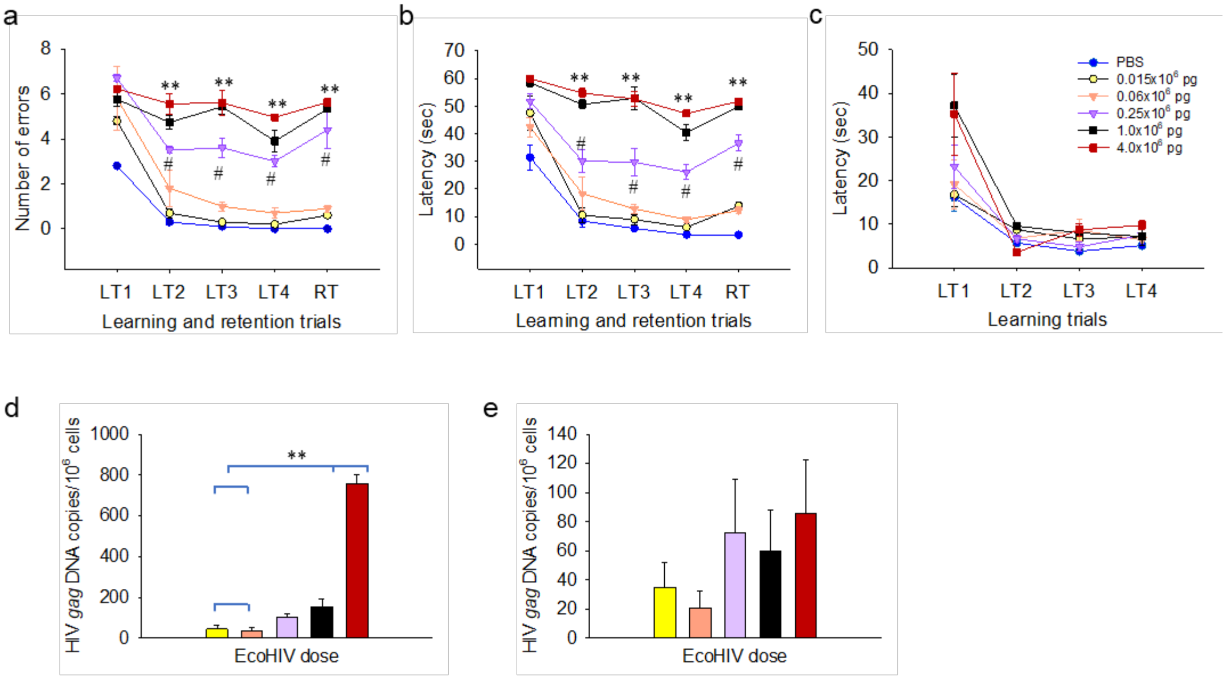

Supplementary Figure S1. Virus dose-dependent learning and memory deficits in mice infected with EcoHIV. Mice in groups of 10 were infected with the indicated EcoHIV dose/mouse or inoculated with PBS and tested in RAWM over 7 days starting at 25 days after infection. (a) Mean number of errors ± SEM made in finding the submerged platform on the last three days of RAWM testing. (b) Mean latencies ± SEM made on the last three days of RAWM testing to find the platform. (c) Mean latencies ± SEM to find visible platform. LT1-LT4 denotes learning trials and RT denotes retention trial performed in RAWM after completing LT4 and letting mice rest in cages for 30 min. (d) SPC and (e) brain HIV gag DNA burdens 30 days after infection of mice with virus doses indicated in panel (c). *$p \leq 0.05$; **$p \leq 0.001$.

(a) For errors: $F_{(5,90)} = 5.489$, $p < 0.001$ (Two-way RM-ANOVA, Bonferroni's post hoc)

$F_{(5,90)} = 46.531$, $p < 0.001$/LT2, $F_{(5,90)} = 49.209$, $p < 0.001$/LT3, $F_{(5,90)} = 55.667$, $p < 0.001$/LT4, and $F_{(5,90)} = 78.579$, $p < 0.001$/RT (One-way RM-ANOVA, Bonferroni's).

(b) For time: $F_{(5,90)} = 8.118$, $p < 0.001$ (Two-way RM-ANOVA, Bonferroni's)

$F_{(5,90)} = 57.774$, $p < 0.001$/LT2, $F_{(5,90)} = 75.691$, $p < 0.001$/LT3, $F_{(5,90)} = 47.835$, $p < 0.001$/LT4, and $F_{(5,90)} = 77.839$, $p < 0.001$/RT (One-way RM-ANOVA, Bonferroni's).

(c) For visible platform: $F_{(5,90)} = 1.163$, $p = 0.263$ (Two-way RM-ANOVA, Bonferroni's)

$F_{(5,90)} = 1.581$, $p = 0.088$/LT2, $F_{(5,90)} = 0.881$, $p = 0.597$/LT3, and $F_{(5,90)} = 0.787$, $p = 0.703$/LT4 (One-way RM-ANVOA, Bonferroni's).

(d) SP gag DNA virology: $F_{(5,30)} = 30.414$, $p < 0.001$ (Two-way ANOVA, Holm-Sidak's)

(e) Br gag DNA virology: $F_{(5,30)} = 1.987$, $p = 0.173$ (Two-way ANOVA, Holm-Sidak's)

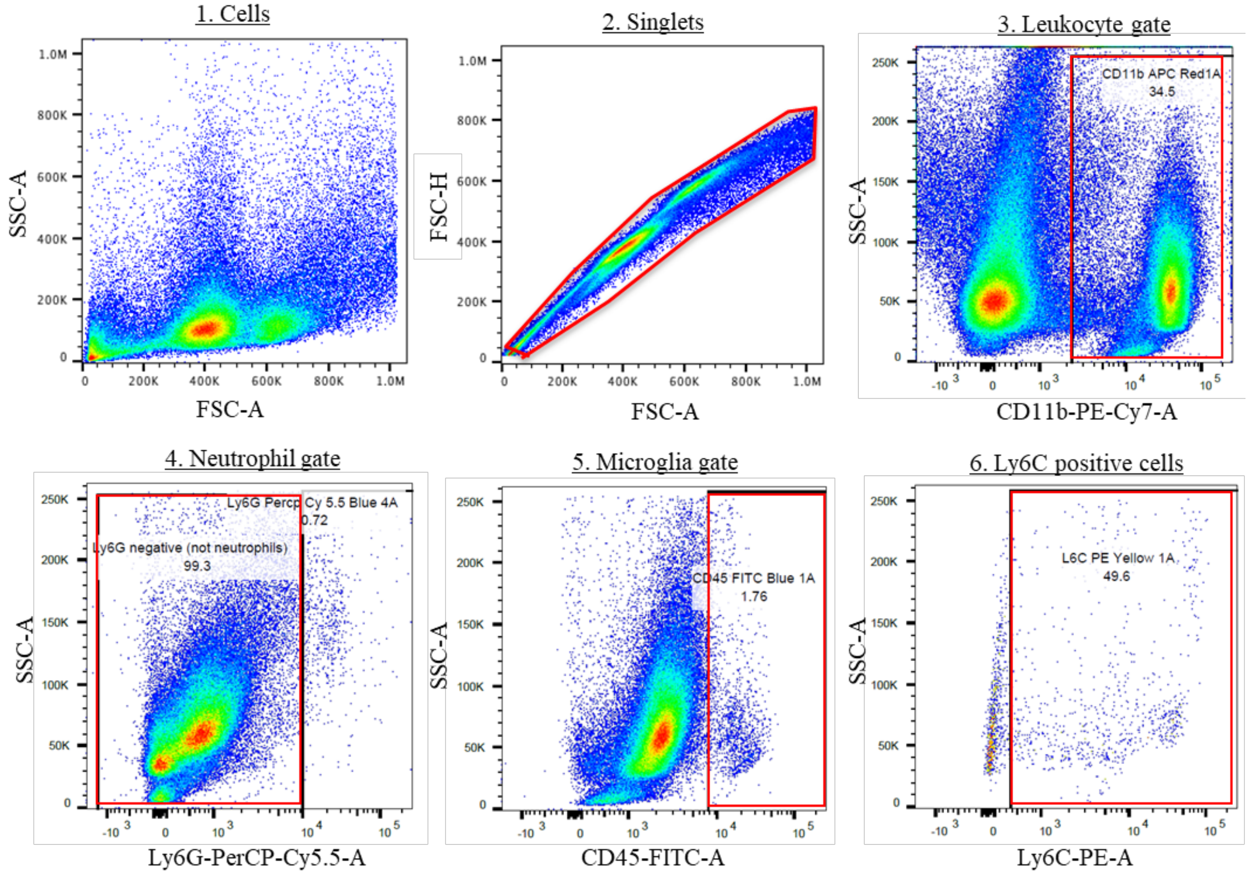

Supplementary Figure S2. The composition of isolated mouse brain immune cells as determined by flow cytometry (n=18/group). Gating strategy to identify and quantify inflammatory brain monocytes. Red gates contained cells analyzed for sequential gating steps. Leukocytes were gated using SSC-A and $CD11b^{+}$. Ly6G negative neutrophils and CD45 high positive microglial cells were excluded from analysis. Inflammatory monocytes were defined as $CD11b^{+}Ly6G^{neg}CD45^{high}Ly6C^{+}$ cells. All gates were determined using the appropriate FMO controls.
